# Cognitive Processing Impacts High Frequency Intracranial EEG Activity of Human Hippocampus in Patients With Pharmacoresistant Focal Epilepsy

**DOI:** 10.3389/fneur.2020.578571

**Published:** 2020-10-27

**Authors:** Jan Cimbalnik, Martin Pail, Petr Klimes, Vojtech Travnicek, Robert Roman, Adam Vajcner, Milan Brazdil

**Affiliations:** ^1^International Clinical Research Center, St. Anne's University Hospital, Brno, Czechia; ^2^Department of Neurology, Faculty of Medicine, Brno Epilepsy Center, St. Anne's University Hospital, Masaryk University, Brno, Czechia; ^3^Institute of Scientific Instruments, The Czech Academy of Sciences, Brno, Czechia; ^4^Behavioral and Social Neuroscience Research Group, CEITEC - Central European Institute of Technology, Masaryk University, Brno, Czechia; ^5^Department of Sports Medicine and Rehabilitation, Faculty of Medicine, St. Anne's University Hospital, Masaryk University, Brno, Czechia

**Keywords:** pharmacoresistant epilepsy, high frequency oscillation (HFO), interictal epileptiform discharge, functional connectivity, hippocampus, cognitive processing

## Abstract

The electrophysiological EEG features such as high frequency oscillations, spikes and functional connectivity are often used for delineation of epileptogenic tissue and study of the normal function of the brain. The epileptogenic activity is also known to be suppressed by cognitive processing. However, differences between epileptic and healthy brain behavior during rest and task were not studied in detail. In this study we investigate the impact of cognitive processing on epileptogenic and non-epileptogenic hippocampus and the intracranial EEG features representing the underlying electrophysiological processes. We investigated intracranial EEG in 24 epileptic and 24 non-epileptic hippocampi in patients with intractable focal epilepsy during a resting state period and during performance of various cognitive tasks. We evaluated the behavior of features derived from high frequency oscillations, interictal epileptiform discharges and functional connectivity and their changes in relation to cognitive processing. Subsequently, we performed an analysis whether cognitive processing can contribute to classification of epileptic and non-epileptic hippocampus using a machine learning approach. The results show that cognitive processing suppresses epileptogenic activity in epileptic hippocampus while it causes a shift toward higher frequencies in non-epileptic hippocampus. Statistical analysis reveals significantly different electrophysiological reactions of epileptic and non-epileptic hippocampus during cognitive processing, which can be measured by high frequency oscillations, interictal epileptiform discharges and functional connectivity. The calculated features showed high classification potential for epileptic hippocampus (AUC = 0.93). In conclusion, the differences between epileptic and non-epileptic hippocampus during cognitive processing bring new insight in delineation between pathological and physiological processes. Analysis of computed iEEG features in rest and task condition can improve the functional mapping during pre-surgical evaluation and provide additional guidance for distinguishing between epileptic and non-epileptic structure which is absolutely crucial for achieving the best possible outcome with as little side effects as possible.

## Introduction

Epilepsy is one of the mostcommon chronic neurological diseases (1) and approximately one third of epileptic patients suffer from a medically intractable form. Those patients are candidates for intracranial EEG (iEEG) monitoring and subsequent surgical treatment of their condition.

The hippocampus is a brain structure that is often involved in temporal lobe epilepsy (TLE). In particular, hippocampal sclerosis is often found in TLE, even though it is not clear whether it is the primary cause of epilepsy, its alteration or consequence (2). Nonetheless, its surgical removal often leads to improvement of the epileptic condition and substantial reduction of seizures (3). The correct determination of epileptic hippocampus and whether the particular hippocampus or its part should be removed can improve the outcome of epileptic surgeries and reduce the unnecessary removal of possible healthy tissue.

In the end of the last millennium, high frequency oscillations (HFO) emerged as a marker of normal function of the brain and epileptic activity (4, 5). Since then, numerous studies have been conducted to evaluate their potential for localization of epileptogenic tissue from iEEG signals (6–11). The distinction of pathological HFO and normal HFO based on their features has been investigated but the results never showed that their separation is possible (12, 13).

The hippocampus is the brain structure where the first HFO were described (4). Physiological HFO in the hippocampus are often studied as markers of cognitive processes and as part of memory formation (14). On the other hand, epileptic hippocampus is often abundant with pathologic HFO (15). It is, therefore, likely that both types of HFO occur simultaneously in epileptic hippocampus and physiological HFO are likely to interfere with the interpretation of the pathological HFO occurrence.

Another iEEG phenomenon connected to epileptogenic tissue and the hippocampus are interictal epileptic discharges (IEDs). They have been proven to be insufficiently specific for the pathological tissue (16), they propagate across multiple brain structures or are generated in zones not generating seizures (green spikes) (17) and can even occur in non epileptic hippocampus (6).

Apart from distinct electrophysiological events such as IEDs and HFO, high frequency functional brain connectivity in ripple and fast ripple frequency range has been used both for studying normal function of the brain and epileptogenic areas (18, 19).

The mentioned high frequency iEEG features represent different underlying electrophysiology. In recent years, the use of machine learning algorithms that combine the diverse information carried by the iEEG features have been shown to outperform the single feature approaches in localization tasks (20–23).

In this study we investigated iEEG features during resting state and task performance to elucidate the impact of cognitive processing on underlying brain electrophysiology under the hypothesis that HFO, IEDs and functional connectivity are modulated differently by cognitive processes in epileptic (EH) and non-epileptic (NEH) hippocampus. The secondary goal of this study was to provide evidence whether these modulations can contribute to better classification of epileptic and non-epileptic hippocampus.

## Materials and Methods

### Subjects

The study was carried out on the data of 36 patients (17 females) with age ranging from 22 to 58 (mean: 37.4 ± 11.3) suffering from medically intractable focal epilepsies. All patients provided a written consent to participate in the study approved by the Ethics Committee of St. Anne's University Hospital in Brno and Masaryk University. Patient information is summarized in Table 1. In most patients, chronic anticonvulsant medication was reduced slightly for the purposes of video-EEG monitoring. All methods were performed in accordance with the relevant guidelines and regulations.

**Table 1:** Study subjects overview with regard to individual hippocampi.

**Analyzed hippocampus**	**Epilepsy side**	**Epilepsy type**	**Engel outcome**	**MRI**	**Histopathology**
Epileptic *N* = 22	Left *N* = 8 Right *N* = 9 Bilateral *N* = 5	Temporal *N* = 22	Engel IA *N* = 12 Engel II-III *N* = 6 NA *N* = 4	Normal *N* = 6 Abnormal *N* = 16	FCD *N* = 3 HS *N* = 8 Negative *N* = 5 NA *N* = 6
Non-epileptic *N* = 23	Left *N* = 12 Right *N* = 11	Temporal *N* = 16 Extratemporal *N* = 7	Engel IA *N* = 10 Engel II-III *N* = 12 NA *N* = 1	Normal *N* = 5 Abnormal *N* = 18	AVM *N* = 1 FCD *N* = 9 HS *N* = 5 Heterotophy *N* = 1 Negatvie *N* = 4 NA *N* = 3

*FCD, focal cortical dysplasia; AVM, arteriovenous malformation; HS, hippocampal sclerosis; NA, not available*.

*Some subjects had both epileptic and non-epileptic hippocampi*.

### Recordings

All patients participating in this study underwent stereotactic depth electrode implantation as part of their presurgical evaluation for treatment of pharmacoresistant focal epilepsy. The localization of the electrodes was determined solely by clinical needs. Used electrodes were either DIXI or ALCIS (diameter = 0.8 mm; inter-contact distance = 1.5 mm, contact surface area = 5 mm^2^; contact length = 2 mm). All used electrodes were MRI compatible. The acquired iEEG was low-pass filtered and downsampled from 25 kHz to 5,000 Hz for subsequent storage and analysis. The used recording reference was the average of all intracranial signals. We analyzed hippocampal stereo EEG (SEEG) during an awake resting interictal period and various simple cognitive tasks.

### Behavioral Tasks

#### Oddball Task

The oddball task was performed similarly to the previous study by Polich (24). Subjects were seated in a moderately lit room with a monitor screen positioned approximately 100 cm in front of their eyes. During the task, they were requested to focus their eyes on the small fixation point in the center of the screen. A standard visual oddball task was performed: three types of stimuli (target, frequent, and distractor) at a ratio of 1:4.6:1, were presented in the center of the screen in random order. The number of targets was 50. Clearly visible yellow capital letters X (target), O (frequent), and various other capital letters (distractor) on a black background were used as experimental stimuli that were presented for 500 ms. The task was divided into four blocks, each block consisted of 12 or 13 target stimuli. The interstimulus interval randomly varied between 4 and 6 s. Each subject was instructed to count the target stimuli in their mind and to report the calculated number after each block.

#### Go/NoGo Task

The Go/NoGo task was replicated from work of Albares et al. (25). Experimental stimuli, i.e., white capital letters A and B, were displayed in the center of the black screen for 0.2 s, followed by a black screen for 2 s. Each letter was preceded by a red or green fixation cross presented with a random duration of 2–6 s. The red fixation cross was followed by the letter A (Go stimulus) or B (NoGo stimulus) with an equal probability. The green fixation cross was always followed by the letter A (Go stimulus). The red cross was twice as common as the green one. In total, 72 NoGo stimuli and 144 Go stimuli were presented, divided into four blocks of the experiment. Participants were instructed to press a button as quickly as possible on Go stimuli and to suppress this action when a NoGo stimulus appeared. Before the experiment, participants completed a short practice.

#### Ultimatum Game Task

The Ultimatum Game task was previously used in an fMRI study by Shaw et al. (26). It presents a simple paradigm to investigate dyadic interaction. The patient was randomly assigned to the role of a Proposer or a Responder. The opposite role was assigned to a nurse willing to participate in the game. Roles were fixed for all rounds.

Each round of the ultimatum game started with the Proposer being given 4 s to choose one of two divisions of a sum of money (of 100 CZK, i.e.,~€4) that differed in the degree of inequity, between themselves and the Responder. After this fixed period, the Proposer's offer was highlighted for 4 s, during which the Responder could either accept or reject the proposal. If they accepted it, then the money was divided accordingly, but if they rejected it, then neither player received any payoff. After this 4-s period, the Responder's decision was then presented for a final 4 s.

The exact same procedure was followed on control rounds, but the choice set comprised two alternative divisions of different colors between the players; rather than dividing a sum of money, Proposers were required to choose the color they preferred for themselves and the color that should go to the Responder, and the Responder then accepted or rejected that offer. Both players were instructed that control rounds had no monetary consequence. Each round ended with a jittered inter-trial interval, with a fixation cross presented pseudo-randomly for 2–4 s. All stimuli were presented to both players simultaneously—Responders saw the initial choice set from which Proposers selected their offer, and Proposers saw the Responder's accept/reject decision. Players were instructed at the start that they would receive the outcome of six rounds selected at random. At no point was any information given to participants on the number of rounds remaining in the task. The whole experiment consisted of two functional runs performed successively in a single session. The two runs together comprised 120 rounds of the experimental condition and 60 rounds of a control condition.

#### Mismatch Negativity

Mismatch negativity (MMN) protocol was based on studies of (27–29).

We recorded a passive task of attention called MMN protocol to find out the presence of MMN/MMN-like response in aiming structures. Each patient lay on the bed in a semi-sitting position with eyes opened. Patient's task was to concentrate voluntary selective attention on watching a self-selected movie and ignore the tones of auditory stimulation, no further information was received. Simultaneously, auditory stimulation was presented binaurally through loudspeakers (~2 m far from ears) in parameters of roving paradigm (frequent and infrequent stimuli).

Frequent and infrequent stimuli (standard and deviant tones of 50/100 ms duration) were randomly presented with the presentation probability of 0.8/0.2. Interstimulus intervals' (ITS) duration was 2,000 ms. All tones were 54 dB (SD ± 4, adjusted subjectively for patient's comfort) SPL, frequency 1,000 Hz, and with jump increase and gradual decrease of the tones' course. The experiment protocol lasted 17 min. This part of investigation was focused on the preattentive detection mechanism on the unconscious level for auditory stimuli which is illustrated by Mismatch negativity.

### Determination of Anatomical Location

To localize the MRI compatible electrode contacts in patients' brains the preoperative MRI was coregistered with postoperative MRI/CT using a custom made Matlab (The MathWorks, Inc.) based on Statistical Parametric Mapping module. After the software coregistration the brain volume was transformed to MNI space and the MNI coordinates of individual contacts were determined. The coregistered volume was used to estimate he anatomical location of each contact by two clinical neurologists using Co-Planar Stereotaxic Atlas of the Human Brain (Talairach-Tournoux system). Only the contacts clearly located in the hippocampus were included in the analysis of iEEG.

### Selection of Hippocampi

The hippocampi in individual patients were classified as epileptic or non-epileptic specifically, according to the results of a standard visual analysis of interictal and ictal SEEG recordings. If contacts implanted in the hippocampus were included in seizure onset zone (SOZ) the hippocampus was classified as epileptic. Conversely, if all contacts implanted in the hippocampus were outside of SOZ and did not exhibit excessive spiking (<50 per 10 min) they were classified as putative non-epileptic hippocampi. The putative non-epileptic hippocampi with spiking above the threshold were visually reviewed whether the IEDs were propagated from other brain structures. The putative non-epileptic hippocampi that generated IEDs were excluded from the analysis.

### Data Processing and Feature Extraction

The iEEG data were processed by automated algorithms that were already used in other published studies. The Python codes of these algorithms are part of the ElectroPhYsiology Computation Module (EPYCOM) and can be found online at https://gitlab.com/icrc-bme/epycom.

#### HFO Detection

The automated detection of HFO was performed by an algorithm used in our previous studies (30, 31). A statistical window of 10 s was used to compute z-scored amplitude envelopes using Hilbert transforms in a series of logarithmically spaced frequency bands (300 bands between 60 and 800 Hz). The detection of putative HFO was done by thresholding the amplitude envelopes by three standard deviations above the mean in each frequency band. The detections overlapping in temporal domain in adjacent frequency bands were joined into one HFO detection obtaining temporal and spectral span of the putative HFO. Final detections were obtained by selecting HFO that have time span >4 cycles at their peak frequency and HFO with minimal frequency at 60 Hz were discarded to remove false positive detections of spikes. HFO amplitude, peak frequency and duration were extracted along with the HFO detections. The detector thresholds were chosen to achieve high sensitivity in order to detect physiological HFO which were shown to have smaller amplitude than pathological HFO (12).

Detected HFO were split into broadband ripple (R; 80–250 Hz) and fast ripple (FR; 250–600 Hz) HFO based on their dominant frequency. Subsequently, HFO rate, mean relative amplitude, duration and dominant frequency per 10 min was calculated for each channel and R/FR and used as features.

#### IED Detection

IED detection was done using the spike detector developed by Barkmeier et al. (32). The detector utilizes filtration in two frequency bands. 20–50 Hz band to detect putative spikes and 1–35 Hz band to determine scaling factor which is used to scale the data in all iEEG channels and to determine amplitude and slope thresholds for final spike detections.

The spike rate and mean spike amplitude per 10 min was calculated for each channel.

#### Functional Connectivity Calculation

Recorded signals were filtered in ripple (80–250 Hz) and fast ripple (250–600 Hz) frequency bands and non-overlapping 1-s sliding windows were used to calculate linear correlation and relative entropy to estimate functional connectivity between iEEG signals recorded by adjacent contacts on an electrode implanted in the hippocampus. For iEEG signals X and Y, the linear correlation was calculated as corr(X,Y) = cov(X,Y)/std(X)·std(Y), where cov stands for covariance and std for standard deviation. The relative entropy was calculated as REN(X,Y) = sum[pX·log(pX/pY)], where pX is a probability distribution of investigated signal and pY is a probability distribution of expected signal.

The connectivity metrics were calculated for R and FR frequency bands and mean value per channel was used in subsequent processing as an iEEG feature.

### Statistical Analysis and Machine Learning

All statistical analyzes and machine learning tasks in this study were performed using custom-made Python scripts, open-source statistical libraries (scipy, statsmodels) and machine learning libraries (scikit-learn).

#### Statistical Analysis

Paired *t*-tests were carried out to evaluate the changes in iEEG features between resting state and during task performance when the patients were under cognitive load for EH and NEH. The statistical difference between EH and NEH during rest and cognitive processing was tested with Mann-Whitney test.

To assess the potential of individual signal features for discrimination of epileptic and non-epileptic hippocampi the receiver operating curve (ROC) and its area under the curve (AUC) was calculated for values during resting state, task performance and for difference of values between resting state and task performance. Hanley-McNeil test was used to determine the ROCs significantly different from chance (AUC = 0.5).

The statistical tests were carried out per channel for each task individually as well as for all the tasks grouped together. In case one subject performed multiple tasks, the mean value of iEEG features across all performed tasks was calculated for statistical testing. To verify that the statistics are not influenced by a subgroup of channels with outlying iEEG features we performed the same analysis per hippocampus where the median of iEEG features from all hippocampal channels was used.

The chosen significance level for all statistical tests was α = 0.05.

#### Machine Learning

The iEEG features with ROC significantly different from chance (AUC = 0.5) either for resting state, task performance or difference between the two states were used to create an SVM model for classification of EH and NEH channels. Only the grouped task ROC values were used for this analysis. To decorrelate the features we used principal component analysis (PCA) during training and testing of the model.

The SVM model was trained and tested in a similar fashion as in our previous work (22) where we performed leave-one-patient-out cross validation for localization of contacts in epileptogenic tissue. Here we use leave-one-hippocampus-out cross validation. The SVM model was trained on all data apart from one hippocampus which was used for classification by the trained model. To optimize the SVM performance, linear and radial basis function kernels were tested and their hyperparameters were tuned by an iterative grid search approach. The performance of the model was evaluated by mean ROC and corresponding AUC calculated from ROCs of each leave-one-hippocampus-out iteration. The evaluated hippocampus was classified as pathologic if the mean probability for classification of the channels as pathologic exceeded 50%. To assess whether iEEG features during rest, cognitive task or the difference between the two states carry different information the SVM model was created separately for each group and for all groups joined.

## Results

### Statistical Analysis

**Figure 1:**
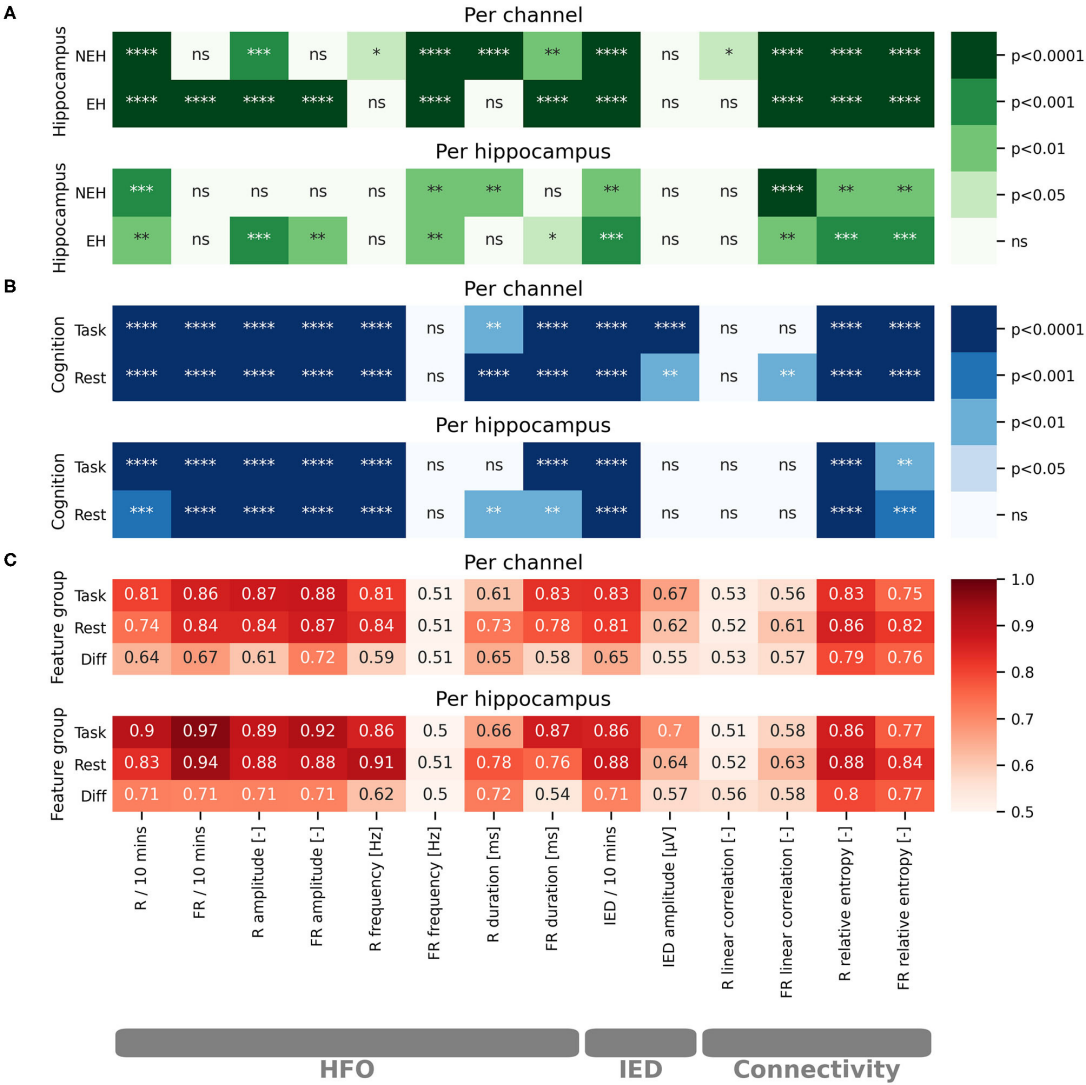
Statistical evaluation of the impact of cognitive processing on iEEG features and evaluation of iEEG feature potential for classification of EH and NEH. The results are visualized for per channel and per hippocampus evaluations. The stars represent the level of significance as marked on the colorbars. Non significant results are marked by “ns.” **(A)** Color-coded paired *t*-test significance level of iEEG features in EH and NEH as a result of cognitive stimulation. **(B)** Color-coded significance between EH and NEH during resting state period and cognitive task. **(C)** Color-coded values of ROC-AUC for classification of EH. ****(*p* < 0.0001), ***(*p* < 0.001), **(*p* < 0.01), *(*p* < 0.05), ns (*p* < 1).

The total number of analyzed channels was 254 (140 EH, 114 NEH) in 45 analyzed hippocampi (22 EH, 23 NEH). The numerical results for all iEEG features are summarized in Table 2 while the results of individual statistical tests are visualized in Figure 1.

**Table 2:** Mean values and standard deviations of iEEG features per channel in EH and NEH channels during rest and cognitive task performance.

**Hippocampus type**	**EH**	**NEH**	**EH**	**NEH**
**Task**	**Rest**		**Cognitive task**	
R/10 min	120.1 ± 141.92	44.94 ± 37.07	64.84 ± 79.77	21.13 ± 14.71
FR/10 min	214.16 ± 327.25	44.28 ± 50.18	137.15 ± 176.33	35.39 ± 24.36
R amplitude [–]	6.87 ± 1.26	5.35 ± 0.93	6.28 ± 1.06	4.95 ± 0.81
FR amplitude [–]	6.62 ± 1.26	5.15 ± 0.86	6.12 ± 0.93	5.05 ± 0.54
R frequency [Hz]	176.75 ± 13.83	153.99 ± 17.42	175.69 ± 11.37	156.96 ± 18.25
FR frequency [Hz]	399.6 ± 28.81	400.05 ± 30.43	412.24 ± 29.2	412.36 ± 22.14
R duration [ms]	34.56 ± 4.13	38.09 ± 4.2	34.41 ± 3.61	35.78 ± 4.19
FR duration [ms]	18.19 ± 2.68	15.11 ± 3.35	17.11 ± 2.69	14.07 ± 1.86
IED/10 min	158.84 ± 154.96	44.81 ± 54.86	105.03 ± 127.74	16.27 ± 31.74
IED amplitude [μV]	378.61 ± 152.44	339.8 ± 172.27	370.88 ± 139.48	320.24 ± 214.9
R linear correlation [–]	0.43 ± 0.21	0.43 ± 0.26	0.44 ± 0.21	0.44 ± 0.27
FR linear correlation [–]	0.49 ± 0.22	0.44 ± 0.2	0.51 ± 0.24	0.48 ± 0.17
R relative entropy [–]	0.29 ± 0.26	0.1 ± 0.05	0.19 ± 0.15	0.08 ± 0.04
FR relative entropy [–]	0.15 ± 0.15	0.06 ± 0.03	0.1 ± 0.08	0.05 ± 0.02

*The statistical evaluation of differences between the values in this table are shown in Figure 1*.

#### HFO

The influence of cognitive processing on HFO was evaluated by comparing the difference in HFO features during resting state and cognitive task performance (Figure 1A). The rate of R was significantly reduced both in EH and NEH as a result of cognitive processing while FR rate was reduced only in EH and remained practically unchanged in NEH. The HFO amplitude was significantly reduced by cognitive processing in EH for both explored HFO groups but in NEH this trend was observed only in the R range. The evaluation of cognitive task influence on HFO duration revealed that the duration was significantly shorter in R band only in NEH and in FR in both NEH and EH. The frequency of HFO in EH and NEH was significantly higher during cognitive stimulation in FR while in R band the significant change occurred only in NEH.

To inspect how HFO features are different between EH and NEH the analysis during resting state and cognitive tasks was performed (Figure 1B).

During resting state, the rate and amplitude of HFO was significantly lower in NEH than in EH in both frequency bands. The duration of HFO in EH compared to NEH was significantly longer in the R band but significantly shorter in the FR band. Significantly lower HFO frequencies in NEH were observed for R band but the difference in FR band was insignificant. During task performance, the HFO rate and amplitude changed similarly to resting state where they were significantly lower in NEH both for R and FR. The duration of R was significantly increased in NEH and, conversely, decreased in FR. HFO frequency during cognitive task was significantly different only in R band, where the NEH exhibited lower HFO frequencies.

The analysis of HFO features utility for classification of EH and NEH was assessed by ROC-AUC during rest, during cognitive task and by the change between the two states (Figure 1C). More than half of the explored HFO features were significantly better than chance (14 out of 24). The HFO rate and amplitude along with R frequency and FR duration showed the highest classification potential both during resting state and task performance.

#### IED

The changes in IED occurrence and amplitude as a result of cognitive task performance was evaluated in a similar fashion as HFO. IED rate was significantly reduced during task in EH and NEH. Conversely, the amplitude of spikes was not influenced neither in EH nor in NEH.

The rate of IED, and IED amplitude were significantly higher in EH during resting state and task performance.

While IED amplitude did not exhibit an ROC significantly better than chance, IED rate reached similar values of AUC as HFO rate and amplitude and was significant for resting state and task performance.

#### Functional Connectivity

The changes in functional connectivity resulting from cognitive stimulation were estimated by linear correlation and relative entropy in the R and FR band. Linear correlation significantly increased during cognitive task in NEH in the R band. In the FR band the significant increase was observed in EH and NEH. The effect on relative entropy was reversed as it was significantly decreased in both bands and hippocampus types.

During resting state, linear correlation was significantly increased in EH compared to NEH only in the FR band while relative entropy was increased in both frequency bands. During cognitive task, relative entropy remained significantly increased in EH but linear correlation did non exhibit any significant difference between EH and NEH.

Hippocampus classification ROC-AUC of linear correlation was slightly higher in FR range but the ROCs were not significantly different from chance. On the other hand, relative entropy showed similar performance as HFO rate and amplitude with highly significant ROCs.

Per hippocampus analysis yielded similar results to per channel bases (Figure 1) with some tests showing nonsignificant results where per channel results were significant. This is a natural effect of performing statistical tests on fewer samples.

### Machine Learning

**Figure 2:**
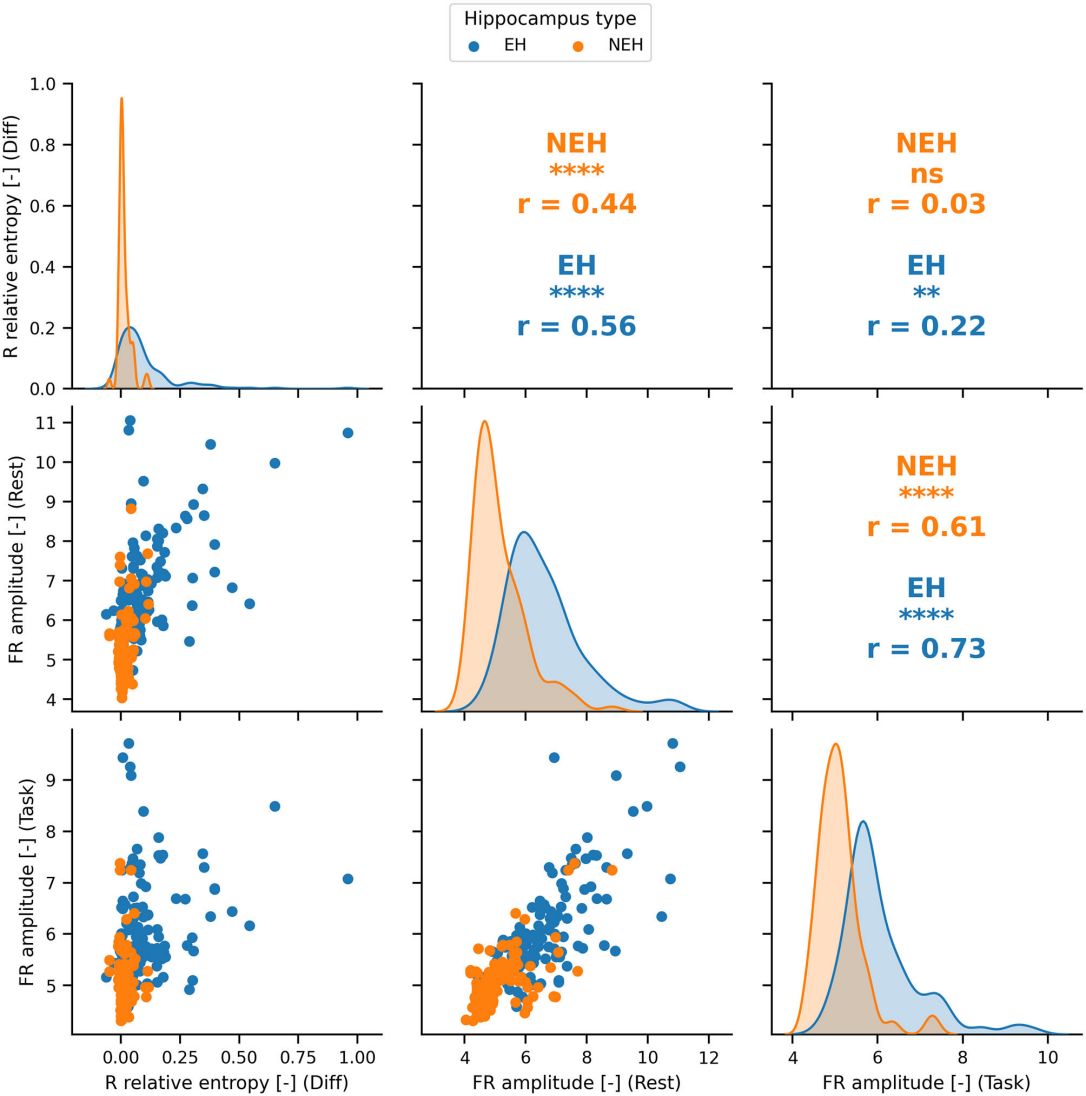
The distributions and Pearson correlation coefficient (*r*) of the best performing features in rest, cognition and the difference between the two states. The best performing features are significantly correlated (significance denoted by stars) in most cases apart from FR amplitude during task and R relative entropy difference in NEH. PCA was therefore used to obtain uncorrelated principal components. ****(*p* < 0.0001), ***(*p* < 0.001), **(*p* < < 0.01), *(*p* < 0.05), ns(*p* < 1).

The features with ROC significantly different than chance during rest, task or the difference between the two states were chosen for the SVM model creation (Figure 1C). The top performing features and their correlation is presented in Figure 2.

The best performing SVM model hyperparameters were determined by an iterative grid search approach (Table 3). This approach was performed for iEEG features during rest, during task performance, the difference between the two states and for all feature groups joined.

**Table 3:** Best performing SVM hyperparameters for individual groups of features and for their aggregate.

**Group**	**Kernel**	**C**	**Gamma**	**AUC**
Only rest	Linear	0.001	–	0.90
Only task	Linear	0.001	–	0.92
Only diff	rbf	0.1	10	0.79
All	rbf	0.1	0.01	0.93

ROC-AUC for classification of EH and NEH channels was calculated for each feature group. The lowest AUC was revealed for rest-task feature differences, followed by features during resting state and task performance. Combination of all features resulted in the highest AUC.

## Discussion

Functional brain connectivity is commonly characterized by activity synchronization of neuronal subpopulations. Widespread neuronal networks including studied hippocampus are thought to be coordinated into synchronous oscillations, HFO during cognitive phenomena but also pathologic epileptic processes. In the presented study we investigated how the iEEG features are influenced by cognitive processing in EH and NEH. We subsequently used the results of this analysis to create an SVM model for classification of channels as EH and NEH.

The higher HFO rate and amplitude in EH during rest and task suggest the possible absence of pathological HFO in NEH and corroborates the results of previous studies (6, 12, 13, 33, 34). Higher resting state R frequency in EH compared to NEH is likely the result of imperfect labeling of FR as R due to the strict frequency boundary of 250 Hz and thus reflects the presence of pathological FR in EH. Some authors have put forward a hypothesis that pathological ripples are only slower fast ripples (11). In NEH, the longer R duration during rest and task performance is not surprising (35, 36). Nevertheless, these results contradict other previously published results (6, 12). This discrepancy might be caused by the fact that the work of Matsumoto et al. was mainly focused on motor cortex which might produce physiological HFO exhibiting disparate features from those in the hippocampus due to histologically different underlying tissue. Conversely to R, FR were longer in EH both during resting state and cognitive task performance reflecting the presence of pathological oscillations (12).

Cognitive processing induced reduction of HFO rates in EH and NEH across all explored frequency bands apart from FR in NEH. The observation that cognitive processing causes R rate decrease and no change in FR in NEH could be the result of decrease in number of R and increase of FR rates observed by Kucewicz et al. (30) in multiple structures including the hippocampus. As other studies previously suggested (37, 38), we hypothesize that the decrease of HFO rate and amplitude in EH as a result of cognitive processing is caused by suppression of epileptic activity in this structure. HFO changes within affected structures may suggest an increased involvement of the preserved normal hippocampal neurons that are active in some physiological cognitive processing and a reduced involvement of the synchronously bursting neurons within the epileptic network that are generating pathological HFO (38). The same explanation can be applied to similar results of possible pathologic ripple reduction in EH. In contrast to EH, the suppression of R rates and amplitude in NEH might be caused by shift of general HFO frequency toward FR band and, therefore, reduction of HFO amplitude and rate. This shift is further supported by the increased R and FR frequency along with shorter R and FR duration in NEH. It is likely that some residual physiological function remains in EH and the effect of reduction of epileptic activity is mingled with the shift observed in NEH.

IED rate was influenced in a similar way as R, being significantly higher in EH during rest as well as during cognitive task and decreased during cognitive task in both types of hippocampus which might reflect the suppression in epileptic activity not only in the hippocampus but also in non hippocampal structures from which the IEDs propagated to NEH. As was shown, specific tasks can suppress focal discharges over the brain regions that mediate the cognitive activity in question (37). IED amplitude was higher in EH than in NEH for both states which is an expected result.

Increased FR linear correlation in resting state EH could be ascribed to functional isolation of epileptic tissue as previously reported (18, 39). The increase in local FR linear correlation during cognitive task likely reflects high neuronal synchronization which is manifested through increased rate of FR HFO (30). Conversely to linear correlation, relative entropy was shown in our previous studies to reflect pathological processes (22, 23). This effect is further confirmed by the results in this study. Decrease in relative entropy during cognitive task further supports the hypothesis that cognitive processing suppresses pathological activity in the brain.

The AUC for classification of NEH and EH using resting state features in an SVM showed good performance. The task performance shower slightly higher AUC suggesting that the changes occurring during cognitive stimulation might carry unique information for localization of hippocampal epileptogenic tissue. The highest AUC was achieved when the SVM model was created with a combination of rest, task and difference features.

We show statistically different electrophysiological reactions of epileptic and non-epileptic hippocampus, which can be measured by HFO, IED and functional connectivity. We propose a hypothesis that cognitive processing reduces pathological electrophysiological activity in EH. Whether this effect is tied directly to stimuli presented to the patient and whether it is present in other brain structures remains to be explored. Analysis of the computed iEEG features in rest and task condition can improve functional mapping during pre-surgical evaluation and provide additional guidance for distinguishing between epileptic and non-epileptic structure which is absolutely crucial for achieving the best possible outcome with as little side effects as possible.

## Limitations of the Study

The NEH classification is problematic because even though such hippocampus is outside of the epileptogenic zone it is still likely influenced by epileptic networks and might exhibit traces of pathological behavior. The influence of different anti-epileptic drugs on the results could not be analyzed due to many variations in medication of individual patients.

## Data Availability Statement

The datasets generated for this study are available on request to the corresponding author.

## Ethics Statement

The studies involving human participants were reviewed and approved by Ethics Committee of St. Anne's University Hospital in Brno. The patients/participants provided their written informed consent to participate in this study.

## Author Contributions

JC carried out the statistical analyses and result visualizations. JC, MP, PK, VT, and MB participated on collection of metadata, writing of the manuscript and interpretation of the results. RR and AV provided data and metadata for cognitive task and assisted in interpretation of the cognitive task results. All authors contributed to the article and approved the submitted version.

## Conflict of Interest

The authors declare that the research was conducted in the absence of any commercial or financial relationships that could be construed as a potential conflict of interest.

## Abbreviations

iEEG: intracranial EEG
TLE: temporal lobe epilepsy
HFO: high frequency oscillation
IED: interictal epileptiform discharge
EH: epileptic hippocampus
NEH: non- epileptic hippocampus
SEEG: stereo EEG.
